# Synthesis and applications of 1D and 2D nanoparticles prepared through crystallisation-driven self-assembly

**DOI:** 10.1039/d6sc01312k

**Published:** 2026-05-29

**Authors:** Kaixiang Yang, Maria C. Arno

**Affiliations:** a School of Chemistry, University of Birmingham, University Rd West Edgbaston Birmingham B15 2TT UK; b Institute of Cancer and Genomic Sciences, University of Birmingham University Rd West, Edgbaston Birmingham B15 2TT UK

## Abstract

Controlled, hierarchical assembly across length scales is a hallmark of natural materials and is often cited as the origin of their desirable properties – bone is the archetypal example, with its impressive combination of stiffness and toughness. Chemists have become adept at manipulating molecules, so it is now a fundamental goal of materials science to achieve a similar level of control over shape and size at the next level up: the nanoscale. This goal is now beginning to be realised, enabled by advances in precision polymer self-assembly methodologies. Crystallisation-driven self-assembly (CDSA) has emerged as a powerful technique to achieve nanoparticles of controlled morphology and size, which chemistry can be manipulated to achieve tuneable properties. In this perspective, we highlight the different methods for the CDSA of anisotropic nanoparticles (1D and 2D) with exquisite control over morphology and dimensions. We discuss the properties of these materials in a variety of different areas, from optoelectronics and information storage to biological processing and materials engineering, illustrating how nanoparticle chemistry can be modulated through living CDSA to produce nanomaterials with unique functionalities.

## Abstract 2

CDSA enables precision growth of 1D fibres and 2D platelets with tunable dimensions and internal structure. This perspective highlights current and emerging applications by linking controllable growth architecture to function and translation.

## Graphical abstract

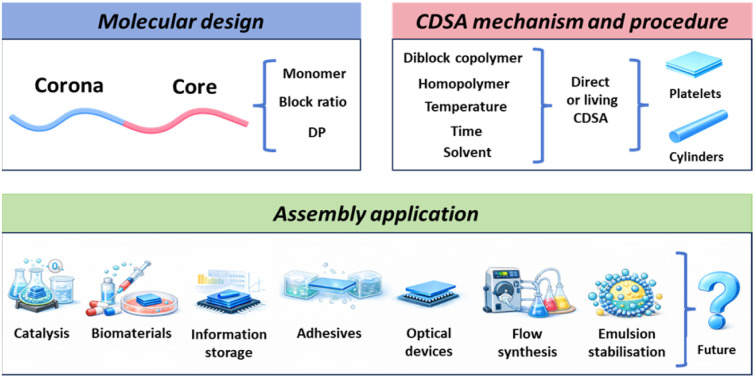

## Introduction

Controlling the self-assembly of synthetic building blocks to access well-defined functional materials on a scale from nanometres to microns remains a key challenge in materials science and nanotechnology.^1–3^ By contrast, Nature routinely achieves exquisite control within this size range through a bottom-up assembly of smaller building blocks to produce monodisperse nanoparticles. Among these, microtubules assembly, cell membranes, and protein quaternary structures are just a few examples of how nanostructure assembly dictates precise function.^4^ Synthetically, amphiphilic block copolymers (BCPs) have been exploited to obtain core–shell micellar assemblies using a solvent that is selective for either the inner (hydrophobic) core or the outer (hydrophilic) corona. However, the formation of anisotropic (*i.e.*, non-spherical) nanoparticles using BCPs is challenging, with only a limited range of amorphous BCP compositions and self-assembly conditions enabling the formation of anisotropic morphologies.^5–7^ This is mainly a consequence of the kinetically trapped nature of BCP nanoparticles, where exchange of block copolymer unimers with micelles is very slow, hence making it difficult to predict the resulting morphology. Moreover, when anisotropic morphologies can be accessed, they are normally a mixture of different shapes rather than a pure form, and no control over nanoparticle dimensions is typically achieved. Nevertheless, several studies have reported that anisotropic morphologies are preferred over their spherical counterparts in a wide range of applications, from therapeutic delivery, to adhesion, and nanocomposites.^8–11^

Solution self-assembly of amphiphilic block copolymers is a widely used route to yield polymer nano-objects with tunable size, internal structure, and surface chemistry. In selective solvents, such systems typically form spheres, worms, rods, or vesicles. Although this approach is broadly applicable and synthetically versatile, reliable access to anisotropic morphologies is often more limited, with low-curvature 1D and especially 2D structures commonly requiring relatively narrow formulation windows and being sensitive to kinetic trapping or mixed-morphology formation.^5^

Other routes to anisotropic polymer nanomaterials have also developed rapidly. Conventional amorphous block copolymer self-assembly can provide 1D structures, while polymerization-induced self-assembly (PISA) has emerged as a particularly powerful and scalable platform for preparing worm-like nanomaterials in relatively large quantities.^7,12^ More recently, branched and bottlebrush-like block copolymer architectures have opened access to non-crystalline 2D nanostructures such as nanodiscs.^13^ Molecular polymer bottlebrushes also represent a complementary platform for 1D polymer materials with overlapping functional and application spaces, particularly in nanomedicine and related soft-material contexts.^14^ These advances collectively highlight both the vibrancy of the field and the continuing importance of improving control over nanoparticle morphology, dimensions, and internal organisation.

Continuing limitations in morphology control, dimensional uniformity, and structural precision have prompted chemists to identify new routes to access nanomaterials with more defined and predictable properties. In 2001 Winnik and Manners reported the first example of crystallisation-driven self-assembly (CDSA), where the semi-crystalline nature of the core block is essential to direct the self-assembly through a crystallisation process.^15–17^ The key role of the crystallisable block was first demonstrated through the use of poly(ferrocenyldimethylsilane) (PFDMS), which was employed to obtain cylindrical micelles in *n*-alkane solvents.^12,18,19^ Since this first report, CDSA has been rapidly established as a powerful tool to achieve anisotropic nanostructures with exceptional control over shape and size.^20,21^ The plethora of polymers used for this technique has also significantly expanded, to include not only further organometallic cores, such as poly(ferrocenylmethylsilane) (PFMS),^22^ poly(ferrocenyldiethylsilane) (PFDES),^20^ poly(ferrocenyldimethylgermane) (PFDMG),^23^ but also biodegradable and biodegradable cores, such as poly(*l*-lactic acid) (PLLA),^24^ poly(caprolactone) (PCL),^25^ poly(spiro[fluorene-9,5′-[1,3]-dioxan]-2′-one) (PFTMC),^26^ poly(δ-valerolactone) (PVL),^27^ poly(ζ-heptalactone) (PHL),^28^ poly(η-octalactone) (POL),^29^ and poly(λ-dodecanolactone) (PDDL).^30^ While the above polymers have been used primarily for applications as drug delivery systems, catalysts, and emulsion stabilisers, π-conjugated polymers such as polythiophene, poly(dihexylfluorene) (PDHF), polyacetylene, and oligo(*p*-phenylene) to name a few, have been exploited for their optoelectronic properties. Without being exhaustive, this list provides an idea of the breadth of research that has undergone in the field of CDSA over the past 20 years, particularly owing to the versatility of this technique and the possibility to predict, and hence tune, nanoparticle morphology and size based on a series of parameters (*e.g.*, temperature, solvent composition, block length). This review will focus on the recent developments in the field, with particular attention to the emerging applications of this technique, as well as attempts to scale up the synthesis of 1D and 2D nanomaterials.^11,20,31^

## Synthetic approaches

This section provides an overview of the different CDSA methodologies available to obtain 1D and 2D nanoparticles, highlighting the strengths and challenges of each method.^32^

### Direct CDSA of block copolymers

Direct CDSA is a straightforward assembly process that operates in two steps: (i) complete dissolution of the block copolymer, which requires melting of the semi-crystalline core, and (ii) cooling and spontaneous nucleation.^15,33^ The main aspect governing self-assembly and crystallisation processes is the solubility of the block copolymer in the assembly solvent.^18,34,35^ Specifically, the assembly solvent does not need to be an excellent solvent for both blocks. Instead, direct CDSA generally requires a solvent (or solvent mixture) in which the crystalline core-forming block is sufficiently soluble when heated above its melting temperature but becomes poorly soluble upon cooling, while the corona-forming block remains solvated over the full temperature range. Hence, a solvent that can solubilise both blocks may suppress CDSA, whereas a solvent that does not fully solvate the corona block may lead to aggregation, phase separation, or precipitation.^36,37^ Computational methods have been used to aid with solvent optimisation, specifically looking at comparing the corona block log *P*_oct_/surface area (SA) value, a theoretical parameter linked to the block hydrophobicity, to the log *P*_oct_ values of a variety of solvents. The difference between corona block log *P*_oct_/SA and solvent log *P*_oct_ was then used as an indicator of solvent quality for the CDSA process. Using this approach, O'Reilly and coworkers identified that ethanol more closely resembled the hydrophobicity of poly(dimethylacrylamide) (PDMA) compared to *n*-propanol, *n*-butanol, and methanol.^38–40^ Consistent with log *P*_oct_ analysis, experimental results confirmed that more well-defined structures could be obtained in ethanol compared to the other solvents.

Despite solvent choice being a key parameter to consider for CDSA processes, other factors, discussed below, need to be considered.

### Influence of the BCP core

The crystalline structure of BCP crystals can strongly influence core shapes. Polymers usually exhibit regular polygonal and geometrical shapes, depending on their lattice structure and crystallisation conditions.^41–43^ The macroscopic morphology of BCPs may be similar to that of homopolymer crystals, when the amorphous block is short. For example, crystals of poly(ferrocenylsilanes) (PFS) are rectangular, poly(ethylene oxide) (PEO) and PCL crystals are square and hexagonal, respectively, while poly(ethylene) (PE) and PLLA form lozenge lamellae.^16,25,36^ Because macromolecular chains are slower to arrange compared to small molecules, as a consequence of the long chain entanglement that constrains chain movement, high crystallisation temperature (*T*_c_) and longer crystallisation times are generally required to allow sufficient reorganisation of polymer chains.^33^ However, not only the core but also the hydrophilic corona is important to the stability and crystallisation of BCPs in different solvents. Representative studies demonstrate that corona solubility, corona dimensions, and solvent quality can strongly influence the morphology and dimensions of nanostructures obtained through CDSA, although the precise effect is system-dependent. Inam *et al.* showed that increasing the corona-to-core ratio in PLLA-*b*-PDMA favored the formation of large 2D diamond-shaped platelets as a consequence of the increased solubility of the PDMA corona in ethanol, whereas decreasing the PDMA corona length led first to mixed platelet/cylindrical morphologies and then to solely cylindrical fibers.^44^ They further demonstrated that increasing corona solubility by introducing a single acid end group shifted the morphology from 1D to 2D, irrespectively of the shorter corona length.^44^ Similarly, Song *et al.* showed that a PFS block copolymer bearing an amphiphilic corona-forming block formed long ribbon-like micelles in isopropanol, mixed platelet/fiber structures in hexanol, and uniform 2D rectangular or oval platelets in octane or octane/hexanol mixtures. The authors concluded that these morphology changes were most likely a consequence of changes in corona block solvation, with hydrogen-bonding and solvent polarity affecting corona dimensions and thereby the assembly outcome.^35^ These examples indicate that corona solvation is an important determinant of whether 1D, 2D, or mixed morphologies are obtained, but that the specific trend depends on the balance between corona solvation, core crystallisation, and overall copolymer/solvent interactions. It is worth noting that if the corona has extremely poor solubility, the corona and core may simply phase separate, leading to irregular assembly or even polymer precipitation.^18,35,45,46^

### Influence of the corona and block ratio

While in the previous section we discussed the shape that BCP crystals can adopt based on the hydrophobic crystalline core, the macroscopic morphology of BCP crystals may also be influenced by the amorphous block, particularly if this is long. For instance, PCL-*b*-[2-(dimethylamino)ethyl methacrylate] (PDMAEMA) BCPs can form spindle-like structures, different from the hexagonal shape of PCL homopolymer crystals.^36,41^ Even though the nanostructures retain the macroscopic morphology of corresponding homopolymer single crystals, their aspect ratios may be different; for example, PCL-*b*-poly(2-vinyl pyridine) (P2VP) BCPs formed elongated hexagonal single crystals.^18,20,34^ Manners and co-workers systematically studied the effect of the amorphous block on the aspect ratio of hexagonal and lozenge single crystals of PCL-*b*-poly(cobaltocenium amidoethyl methacrylate) (PCoAEMA). They found that the crystals' aspect ratio decreased linearly with increasing PCoAEMA/PCL block ratio. Tong and co-workers prepared BCPs of PEO-*b*-PCL-*b*-PDMAEMA. Through counterion exchange, various counterions with different hydrophilicity, such as Br^−^, I^−^, SCN^−^, PF_6_^−^ and OTf^−^, were introduced into the amorphous block, enabling control over the aspect ratio of the BCPs.^35,45,47,48^

The length of the crystalline core block also plays a critical role in systems with fixed corona-to-core ratios. O'Reilly and co-workers found that, in good solvent and with a long amorphous block, 2D lamellae tend to be formed, while in poor solvent and with a short amorphous block, rod-like or irregular assemblies appeared. They proposed that, as the BCPs become less soluble, there is no adequate time for the crystalline chains to adopt a preferred conformation, resulting in less defined structures.^18,34,35,39,40^ It is worth noting that polymers with low dispersity are required in order to obtain crystalline assemblies of uniform size and shape, as the molecular weights of both the crystalline and amorphous blocks strongly affect the solubility and the ability of the BCPs to crystallise, as well as the chain folding number of the crystalline block.^49^ Therefore, living and controlled polymerisations, such as anionic polymerisation, atom transfer radical polymerisation (ATRP), reversible addition–fragmentation chain transfer polymerisation (RAFT), and ring-opening polymerisation (ROP) are usually applied to prepare crystalline BCPs with narrow molecular weight distributions.^2,50^

### The role of the homopolymer

Homopolymer additives can significantly influence block copolymer self-assembly by modifying core swelling, chain packing, and the balance between interfacial energy and chain stretching. Early work by Eisenberg and co-workers showed that, in blends of polystyrene-*b*-poly(acrylic acid) (PS-*b*-PAA) and homopolystyrene, low homopolymer loadings could be accommodated within the micelle core, whereas higher homopolymer contents led to the formation of larger and structurally distinct crew-cut aggregates. These studies established homopolymer addition as an effective handle for tuning the size and morphology of block copolymer assemblies in solution.^1,51^

In CDSA, homopolymers can play an even more active role by redirecting the assembly pathway itself. Eisenberg and co-workers showed that compatible homopolymers can act as structure-directing agents in semicrystalline block copolymer micelles.^51^ Building on this concept, Manners and co-workers demonstrated that blends of crystallizable homopolymers and block copolymers could be used to access uniform 2D platelet micelles,^52^ while later studies from O'Reilly and co-workers showed that homopolymer/block copolymer blend unimers can promote platelet growth in biodegradable polylactone systems; in contrast, in the absence of homopolymer, growth tends to remain one-dimensional to yield cylindrical structures.^30^ Together, these studies show that homopolymer incorporation is not merely a means of increasing micelle size, but a powerful strategy for directing growth from 1D to 2D in suitable CDSA systems.

### Influence of heating time and cooling temperature

The cooling rate significantly affects the morphology, size, and branching structure of the micelles. Although no universal predictive rule exists based on the melting point of the core-forming polymer alone, cooling rate provides a useful practical handle to predict CDSA outcome. In general, slower cooling allows more time for chain rearrangement and often favours more regular, thermodynamically preferred structures. On the contrary, rapid cooling increases kinetic trapping, nucleation density, and the likelihood of branching, leading to less defined morphologies. Xu and coworkers examined the influence of crystallisation temperature on the morphology of poly(3-caprolactone)-*b*-poly(ethylene oxide) (PCL-*b*-PEO) micelles in aqueous solution.^36,53^ They used water-THF mixtures to dissolve the BCPs and then heated the solution above the melting temperature of the PCL block.^43^ The samples with a shorter PEO corona block formed lamellar micelles when cooled to 20 °C, but cylindrical micelles when cooled to 0 °C. In contrast, the samples with a longer PEO block showed a different behaviour, with lamellar and cylindrical micelles formed at the lower crystallisation temperature. Moreover, Manners and colleagues demonstrated that a rapid cooling rate leads to an increase in micelle size, a transition in morphology, and the formation of branching structures. At a cooling rate of 10 °C h^−1^, the resulting micelles showed an average diameter of about 50 nm and exhibit more branching, forming spherical structures. However, at a cooling rate of 1 °C h^−1^, the average diameter of the micelles increased to approximately 80 nm, and their morphology transitioned from spherical to elongated, with a lower degree of branching and a more regular shape.^54–56^

### Other parameters that affect CDSA processes

In addition to the factors mentioned above, the CDSA of BCP can be influenced by other parameters, such as concentration and phase separation as a consequence of BCP incompatibility. The concentration of the BCP solution determines the interactions between polymer chains and hence the dynamics of self-assembly.^57^ High BCP concentrations may lead to larger structures, owing to more polymer chains joining together. When there is incompatibility between the two segments of a BCP, the system undergoes a rapid phase separation, resulting in more distinct core/shell structures or compact morphologies, such as spherical or cylindrical micelles.^58^ In contrast, better compatibility between segments can promote the formation of more complex structures, such as platelets or network-like assemblies.^56^

### Living CDSA

While the direct CDSA method described earlier can be seen as straightforward, despite the need to control different conditions for it to be successful, the lack of precise control over nanoparticle size poses challenges for applications that require stricter design specifications for nanomaterials. To overcome this issue and achieve nanoparticles of controlled size in both 1D and 2D, Winnik and Manners pioneered the process of living CDSA.^20,59,60^ This technique involves first the fabrication of crystalline seeds from long nanostructures obtained through a direct CDSA process. The seeds were generated through sonication and defined as the shortest nanostructures that can be achieved through this method (*e.g.*, length = 20 nm). Then, the BCP was dissolved in a suitable solvent for both the core and corona blocks to generate unimers, unassembled polymer chains, which were then added to a solution of crystalline seeds. The process was shown to resemble a living polymerisation of molecular monomers, offering a unique strategy to create highly uniform populations of precisely designed 1D (rod-like) and 2D (platelet-like) nanoparticles.^50^ Importantly, by controlling the amount of BCP added to the seed solution, the size of the nanoconstructs can be precisely tuned. Indeed, in a true living CDSA process the theoretical length of 1D nanostructures and the area of 2D platelets can be predicted based on the unimer to seed ratio in solution.^20^ Moreover, by simply changing the chemistry of the BCP unimers, this technology enables to obtain nanostructures with spatially defined chemistries that are challenging to be accessed through more conventional self-assembly methodologies. For example, 1D nanostructures with chemically distinct corona segments can be prepared through the sequential addition of unimers with different corona-forming blocks, achieving assemblies with precisely controlled dimensions.^61,62^ Moreover, 1D triblock nanostructures with segmented cores have been prepared using a heteroepitaxial growth process by the addition of BCP unimers with a crystallizable PFDMG core to seeds with crystalline PFDMS cores. Additionally, segmented and gradient co-micelles have been achieved by the co-assembly of two types of unimers with different growth rates.^61,63,64^

**Fig. 1:**
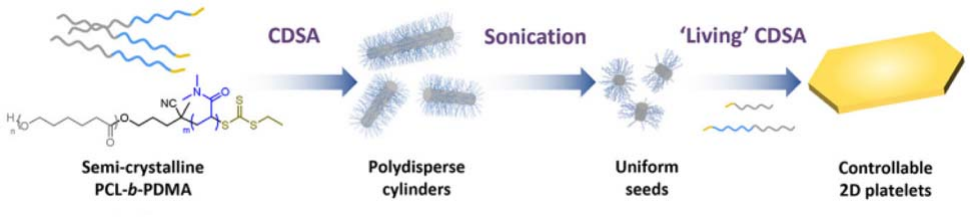
Precise control of 2D platelet growth. Synthesis route for PCL/PCL-*b*-PDMA platelets through a living CDSA approach. Adapted with permission from ref. 65. Copyright 2023. ACS Publications.

Since the proof-of-concept work on living CDSA with PFDMS, other crystallisable polymer systems have been used for a wide variety of applications. Reported studies involved PLLA, PE, PFDES, PCL, PHL, PVL, POL, PDDL, polycarbonate, and π-conjugated polymers such as poly-thiophene, poly(3-heptylselenophene), and PDHF.^20,30,65–68^ With some of these systems (*e.g.*, the polylactones), heteroepitaxial growth has been achieved by the co-crystallisation of compatible polymer cores.^25,69,70^ Moreover, seeded heteroepitaxial growth of the crystallizable polymer blends in two dimensions has been successfully achieved to prepare well-defined platelets.^71,72^ Indeed, multiblock platelets with cores that can degrade at different times were accessed through 2D living CDSA with sequential addition of different unimers.^39^(Fig. 1).

### CDSA in flow

The translation of CDSA from batch protocols to continuous-flow environments represents a methodological shift rather than a simple scale-up strategy.^73^ Traditional batch CDSA excels at producing nanostructures with exceptional uniformity, yet it is intrinsically limited by heterogeneous mixing, poorly defined thermal histories, and sensitivity to operator-dependent parameters. Recent studies demonstrate that continuous flow technologies can directly circumvent these constraints, positioning CDSA for translation from laboratory precision to industrial relevance.^74^ For example, continuous flow has been shown to maintain stable unimer delivery, regulate supersaturation, and support reproducible seeded growth over extended operation windows, enabling long-term production of uniform nanotubes and nanofibers.^75^

Beyond practical considerations of scalability, the use of flow systems revealed new mechanistic regimes of nucleation and growth that are difficult to access in batch. Rapid mixing and finely tuned residence-time distributions enabled early-stage nucleation, as demonstrated by the direct preparation of 2D platelets through fast seed formation under flow-induced supersaturation.^76^ These results shift the role of flow from an engineering convenience to a mechanistic tool capable of dictating crystallisation pathways. This perspective is reinforced by a growing body of flow-CDSA studies reporting access to unusually small, highly monodisperse seeds, previously inaccessible morphologies, and unconventional crystallisation trajectories enabled by controlled hydrodynamics.^77^

The modular architecture of continuous-flow systems further enables hierarchical and multistep CDSA processes. Sequential unimer addition under flow conditions allows the preparation of segmented or multiblock nanostructures with reduced defect incorporation and improved compositional fidelity.^78,79^ Integration with related approaches – such as polymerisation-induced CDSA, inline photochemical activation, or microreactor-assisted nanoparticle deposition – extends this concept toward continuous, programmable assembly pipelines. Coupled with real-time analytical tools, flow CDSA opens the possibility of feedback-controlled crystallisation, a prerequisite for autonomous and self-optimizing assembly platforms.^32^

Looking forward, continuous manufacturing may redefine how CDSA-derived materials enter technological applications, including anisotropic conductors for optoelectronics, aligned catalytic nanofibers for energy conversion, and precision-engineered biomaterials for immunomodulation or tissue interfaces. Achieving these ambitions will require advances in flow-compatible block-copolymer synthesis, predictive crystallisation models capable of operating under non-equilibrium conditions, and downstream integration with formulation and device-fabrication steps. Nevertheless, the trajectory is clear: continuous flow provides a decisive route toward transforming CDSA from a sophisticated laboratory method into a robust, industrially scalable manufacturing platform.

## Unique properties and applications of 1D and 2D nanoparticles

CDSA provides a distinct conceptual framework for polymer self-assembly in which nanostructure architecture is encoded during growth rather than inferred after formation.^80^ In contrast to conventional amphiphilic self-assembly, where anisotropy often emerges statistically, CDSA enforces directional crystallisation that enables dimensionally addressable nanostructures with prescribed length, internal segmentation, and crystallinity. This growth-determined architecture transforms 1D fibers and 2D platelets from morphological outcomes into controllable variables, allowing nanoscale geometry to be directly linked to functional performance.^2,81–84^ This capability is particularly helpful in application spaces where anisotropic nanoparticles are limited by polydispersity, interfacial ambiguity, or uncontrolled defect populations. By minimizing such uncertainties, CDSA-derived nanostructures function not only as materials components, but also as model systems for identifying transferable structure–function relationships. In the sections below, we highlight how this architectural control manifests across optical, mechanical, biomedical, and energy-related contexts.^32,85^

## Optical and photonic functions: growth-programmed information encoding

**Fig. 2:**
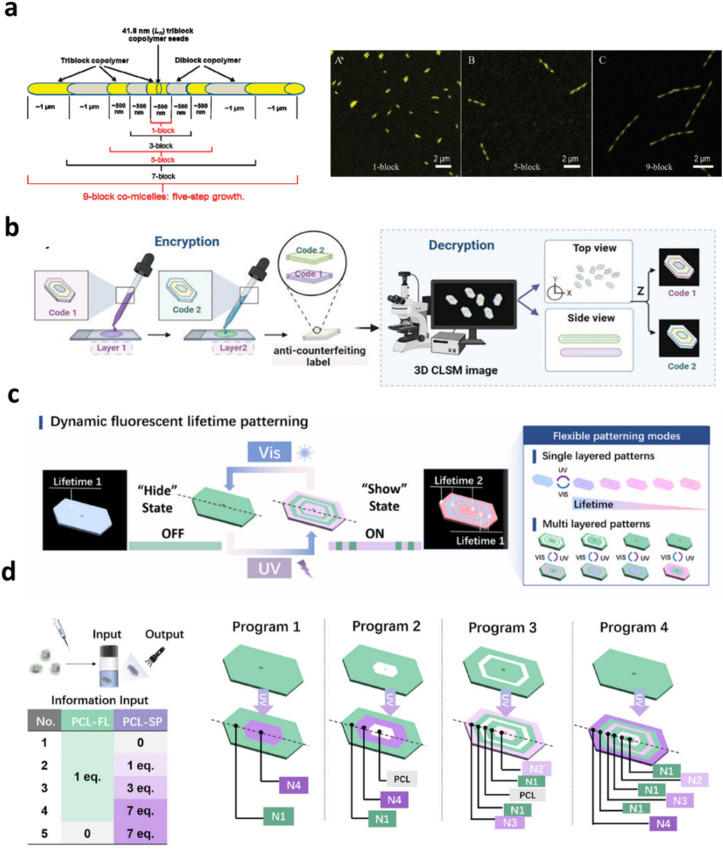
(a) Multiblock one-dimensional micelles formed *via* living CDSA exhibit spatially defined fluorescent segments, allowing optical patterns to be encoded along a single axis as colour-resolved barcodes. Adapted with permission from ref. 61. Copyright 2011. ACS Publications; (b) controlled seeded growth of two-dimensional platelets yields hierarchical microbarcodes with laterally organised, multilayer emissive domains. Adapted with permission from ref. 90; (c) photoresponsive microbarcodes enable temporal control over fluorescence lifetimes, introducing a dynamic dimension to optical information storage; (d) schematic representation of multilayer fluorescent platelet fabrication (Programs 1–4) through sequential incorporation of dye-labelled blending unimers (N1–N4) and non-emissive PCL unimers. Adapted with permission from ref. 91.

Assemblies prepared through CDSA methodologies have emerged as programmable optical materials in which uniform 1D fibers serve as spatially decoded fluorescent elements for information storage.^86–88^ He *et al.* employed epitaxial unimer insertion to generate multiblock micelles with spatially segmented fluorescence, producing barcodes that display sharply banded emission with high contrast and stability (Fig. 2a).^61^ The significance of achieving precise control over the length and internal architecture of these nanostructures through living unimer addition is considerable for information encoding, as such deterministic nanoscale patterning would be exceedingly difficult to accomplish using lithographic strategies.^89^ One-dimensional cylindrical micelles formed *via* living CDSA provide a clear demonstration of this principle. Through sequential unimer addition, fluorescent blocks can be inserted at prescribed positions along the fiber axis with near-quantitative fidelity, yielding multiblock nanostructures with sharply segmented emission domains. These architectures function as nanoscale optical barcodes, where information is encoded through segment sequence and length rather than post-assembly patterning, an approach that is fundamentally distinct from lithographic fabrication.^62^

Two-dimensional CDSA architectures further expand the information landscape by introducing areal, rather than linear, encoding. Xie *et al.* developed a seeded living CDSA method to construct hierarchical 2D microbarcodes with ultrahigh information density encoded simultaneously in colour and geometry (Fig. 2b).^90^ By precisely sequencing polymer deposition, they achieved platelets with spatially resolved, multilayered fluorescence patterns, thereby elevating the conceptual ceiling for encoding capacity. Extending this strategy, Tong, Xie, and colleagues devised “light-flashable” 2D fluorescent lifetime microbarcodes incorporating photoswitchable spiropyrans (SP), enabling dynamic modulation of fluorescence lifetimes *via* energy-transfer switching (Fig. 2c and d).^91^ This innovation adds a temporal dimension – light-gated optical addressability – facilitating multidimensional patterning and improving robustness against noise and data degradation. Together, these developments underline how CDSA merges programmable crystallisation with functional photonics to drive the next generation of high-density and multifunctional optical data storage technologies.^31,92^

Within the expanding landscape of CDSA-enabled optical information systems, the broader context of nanotechnology reveals why such advances are timely and consequential. One of the major challenges in optical data storage lies in achieving precise, large-scale data writing and retrieval in a controllable manner, and nanotechnology is now reshaping how this challenge is conceptualised.^93^ Although top-down nanofabrication strategies such as photolithography and 3D printing have been deployed for nanoscale optical storage, their resolution limits and micro/nanoscale precision impose intrinsic constraints that hinder future gains in storage density and fidelity. In contrast, bottom-up nanofabrication – guided by the thermodynamics of polymer self-assembly – presents an emerging paradigm that decouples feature definition from lithographic restrictions.^94^ These assembly-driven approaches promise to surpass traditional limitations, offering unprecedented accuracy and capacity for optical information technologies. Self-assembled DNA scaffolds and inorganic nanoparticles already demonstrate that nanoscale optical patterns can be generated with exceptional spatial precision, opening new possibilities for high-resolution storage.^95–98^

Within the field of optoelectronics, CDSA offers a particularly compelling opportunity: by using directional crystallisation as a nanoscale architectural principle, it promotes deterministic 1D and 2D epitaxial growth with a degree of structural fidelity and programmability rarely attained in polymer systems.^80^ The integration of multicolour emission and dynamically tuneable fluorescence lifetimes further positions CDSA as a transformative platform for next-generation optical materials.^83,99^

## Mechanical reinforcement and interfacial function: geometry as a design variable

In mechanically functional composites, nanoparticle geometry and interfacial compatibility can be as decisive as chemical composition in determining performance. Conventional fillers often suffer from ill-defined shapes or broad size distributions, limiting their ability to interact predictably with polymer networks. CDSA-derived 1D and 2D nanoparticles overcome these limitations by providing reinforcing elements with precisely defined dimensions and crystalline rigidity.^3,100,101^ Owing to their semi-crystalline nature, CDSA-derived fibres are typically more shape-persistent and less flexible than more deformable cylindrical assemblies, which can be advantageous for reinforcement and structural integrity but may be less desirable where flexibility or adaptive deformation is required.^102^

### Mechanical and adhesive reinforcement

**Fig. 3:**
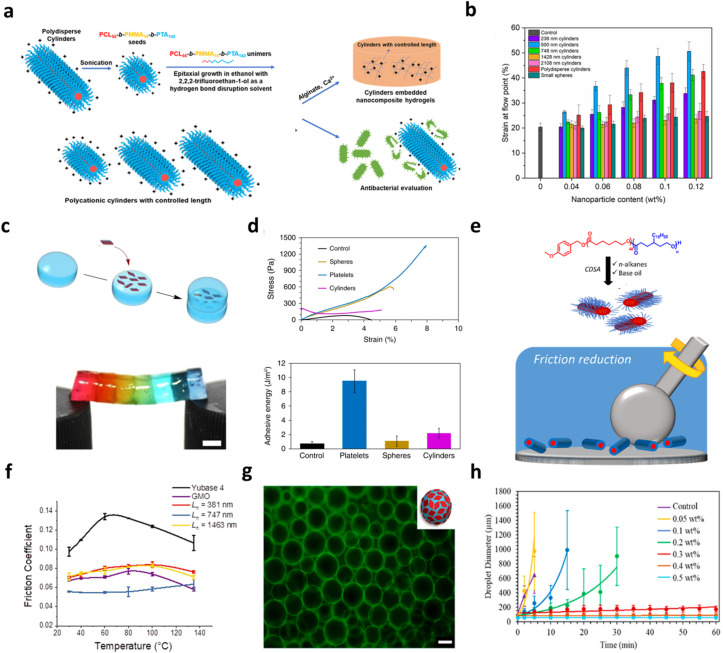
(a) Illustration of the formation of PTA-based polycationic cylindrical micelles with controlled lengths *via* epitaxial growth and their incorporation into cylinder-reinforced nanocomposite hydrogels; (b) histogram showing the strain at the flow point for nanocomposite hydrogels containing varying wt% of cationic cylindrical micelles; error bars indicate standard deviation. Adapted with permission from ref. 105; (c) schematic of alginate hydrogel block adhesion mediated by quaternized platelets, alongside a photograph of calcium-alginate blocks joined using PLLA_35_-*b*-PDMAEMA_315_ platelets suspended horizontally. Scale bar = 0.5 cm; (d) bulk shear stress measurements comparing interfacial stresses and adhesive energies for two identical calcium-alginate gel blocks in contact with water (control), platelets, cylindrical micelles, or spherical micelles. Adapted with permission from ref. 10; (e) illustration of the friction-reducing mechanism of cylindrical micelles in lubricants; (f) comparison of friction coefficients for cylinders of different lengths in Yubase 4, neat base oil, and glycerol monooleate (GMO). Adapted with permission from ref. 110. Copyright 2023. ACS Publications; (g) fluorescence microscopy image of emulsion droplets stabilized by platelets; (h) temporal evolution of emulsion droplet diameters at different platelet concentrations. Adapted with permission from ref. 118. Copyright 2017. ACS Publications.

One-dimensional fibers act as anisotropic load-bearing components whose length can be deliberately matched to characteristic network length scales, such as polymer entanglement distances or crack-bridging zones.^103^ By tuning fiber dimensions through controlled CDSA growth, mechanical reinforcement can be optimized without increasing filler loading, thereby preserving material processability.^104^ The O'Reilly group generated 500 nm cylindrical nanoparticles that were able to significantly enhance (up to 51%) the resistance to break under strain of the hydrogels they were incorporated in, which was higher than their counterparts with different dimensions (Fig. 3a and b).^105,106^ Li and co-workers toughened commercial poly(methyl methacrylate) (PMMA) resin using poly(cholesteryl methacryloyloxy ethyl carbonate) (PCholMA)-based cylinders, and the influence of cylinder length on toughening effect was studied. Results indicated that cylinders around 950 nm in length could achieve the maximum toughening effect while increasing chain flexibility of the matrix.^107^

2D platelets add reinforcement through enhanced interfacial area and shape anisotropy, improving stress transfer and adhesion within soft matrices or at interfaces. For example, the use of PLLA-based nanoparticles with platelet morphology as an adhesive resulted in a significant enhancement of mechanical property and adhesion over nanoparticle glues comprised of spherical or cylindrical micelles (Fig. 3c and d).^10^ Moreover, the biodegradable and biocompatible nature of the materials used provides significant potential for use in a wide range of applications, including tissue engineering and drug delivery.

### Friction modifiers

Anisotropic nanoparticles obtained through CDSA are promising as polymeric friction modifiers in lubrication systems, competing with inorganic additives that often sediment.^108,109^ In a seminal demonstration, Clamor *et al.* prepared cylindrical micelles directly dispersed in lubricant media *via* living CDSA, achieving controlled lengths and excellent colloidal stability. By systematically varying the micelle length, they established a direct relationship between nanofibrer geometry and frictional behaviour, observing that all anisotropic samples contributed to friction reduction relative to neat oil. In particular, cylinders with lengths near 750 nm displayed the greatest decrease in friction, even compared with a common commercial additive, glycerol monooleate, highlighting the promise of CDSA nanostructures for reducing mechanical wear and enhancing energy efficiency in advanced lubrication applications (Fig. 3e and f).^110^

### Emulsion stabilisation

2D particles outperform small molecule and block polymer emulsifiers in stabilising pickering emulsions, with CDSA facilitating the fabrication of platelets with deterministic lateral dimensions for this type of applications.^111–113^ This makes CDSA well-suited for creating hierarchical composites, structured colloids, or supracolloidal assemblies with deterministic architecture.^114–117^ The Dove and O'Reilly groups prepared PLA-based platelets *via* CDSA and used their precisely controlled lateral dimensions to formulate water-in-water emulsions. Fluorescent labelling of the platelet-forming polymers allowed direct visualisation of their localisation at droplet interfaces. Larger platelets produced more robust emulsification than smaller ones, and droplet stability increased with platelet loading, remaining intact for roughly an hour at concentrations above 0.3 wt% (Fig. 3g and h).^118^ More recently, the same team applied PCL-derived platelets to oil-in-oil systems, obtaining emulsions that remained stable for more than four weeks. These biodegradable, biocompatible 2D particles therefore offer a promising route to high-performance emulsifiers for pharmaceutical and cosmetic formulations.^119^

## Biomedical applications of nanomaterials prepared through CDSA

Biological environments are acutely sensitive to nanoscale features such as size, shape, and surface chemistry, making them challenging scenarios for precision biomaterial design.^80,120,121^ By dictating the size, shape, and interfacial chemistry of these assemblies, CDSA directly influences how the resulting nanoparticles interact with cells, circulate within biological environments, and perform in therapeutic contexts. Moreover, CDSA-derived nanostructures feature architecture-resolved design, in which individual parameters can be systematically varied while others are held constant, hence representing excellent nanomaterials for interfacing biological environments.^121,122^

### Cellular-level interactions and immunomodulation

Nanoparticle morphology, size, and surface chemistry dictate endocytosis pathways, intracellular trafficking, and immune recognition, hence directly impacting therapeutic efficiency and safety of nanomaterials.^123–127^ In 2021, Yu and colleagues investigated the cellular uptake and tumour-penetration behaviour of rigid rod-like micelles formed *via* CDSA from a diblock copolymer composed of a crystallizable core and a PEG-based corona. They prepared micelles with a uniform width (∼12 nm) and lengths ranging from 80 to 2000 nm and compared their performance in multicellular tumour spheroids (MCTS) derived from two human breast cancer cell lines (MDA-MB-436 and MDA-MB-231). The nanoparticles exhibited length-dependent tumour spheroid penetration, with shorter rods achieving deeper tissue penetration. These results underscore that both the shape and the precise length of the micelles significantly modulate their cellular uptake and tissue penetration – a behaviour highly dependent on the geometry of the self-assembled nanocarrier.^128^

**Fig. 4:**
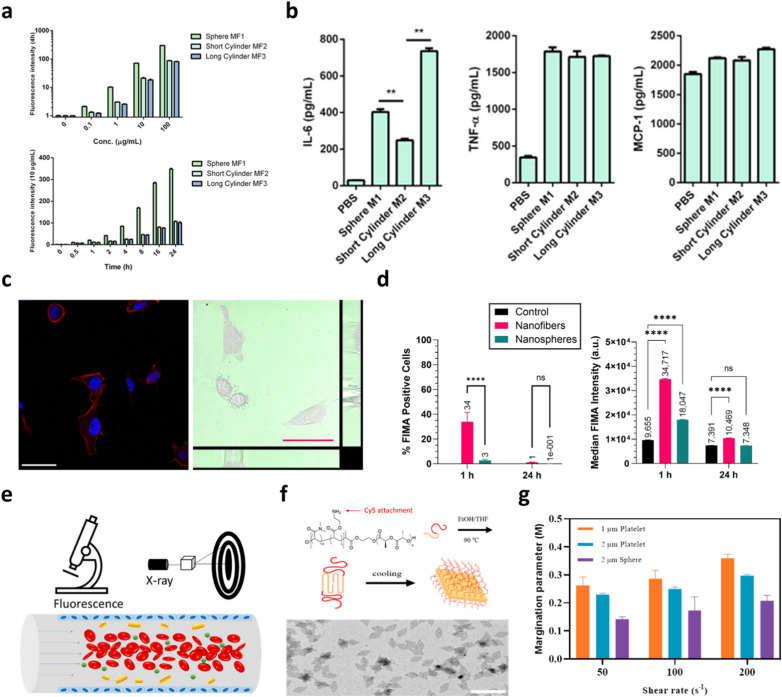
Morphology-dependent biological interactions of CDSA-derived nanostructures. (a) dose- and time-resolved binding profiles of glyco-nanoparticles with RAW 264.7 macrophages, illustrating accumulation kinetics; (b) proinflammatory cytokine secretion (IL-6, TNF-α, MCP-1) by macrophages following 24 h exposure to 10 µg mL^−1^ glyco-nanoparticles, quantified by ELISA. Adapted with permission from ref. 123. Copyright 2016. ACS Publications; (c) confocal laser scanning microscopy (CLSM) of HeLa cells incubated with fluorescent segmented PFTMC-b-PHPMA nanofibers, with nuclei (DAPI, blue), F-actin (Alexa Fluor™ 594 phalloidin, red) and nanofibers (green) shown; right panels depict live-cell brightfield images with nanofiber distribution (scale bar = 50 µm); (d) flow cytometry analysis of U-87 MG glioblastoma cells indicating the percentage of fluorescein-positive cells and median fluorescence intensity after treatment with segmented nanofibers *versus* blended nanospheres. Adapted with permission from ref. 129; (e) schematic illustration of the margination behaviour of platelet-like 2D nanoparticles under blood flow; (f) synthetic scheme and TEM image of representative CDSA-derived 2D platelets; (g) quantitative comparison of margination parameters for 1 µm and 2 µm platelets and 2 µm latex spheres under varying shear rates, highlighting enhanced near-wall enrichment for platelet geometries. Adapted with permission from ref. 8. Copyright 2023. ACS Publications.

Beyond uptake, immunomodulatory responses also depend on shape and size, particularly in vaccine design. In 2016, Li and colleagues exploited how the shape and dimensions of glyco-functionalised nanostructures, prepared *via* CDSA of block copolymers, affect interactions with immune cells. Their data showed that spherical glyco-micelles were internalised by macrophages to a much greater extent than the cylindrical glyco-nanoparticles, likely due to differences in endocytosis pathways and membrane-wrapping kinetics. Interestingly, when assessing inflammatory cytokine production, longer cylindrical glyco-nanoparticles induced a more pronounced secretion of interleukin-6 (IL-6) compared to the spherical counterparts or shorter cylinders, despite their lower uptake. These findings demonstrate that both the morphology (1D *vs.* roughly spherical) and size (length of cylinders) of glyco-CDSA nanostructures critically influence not only cellular internalisation but also the magnitude of immune activation – a key consideration when designing nanocarriers for immunomodulation or therapeutic delivery (Fig. 4a and b).^123^ Together, these studies suggest a future where CDSA assemblies act as quantitative probes, linking structural features directly to cellular responses rather than relying on empirical correlations.

### *In vivo* biodistribution, circulation, and biodegradation

The pharmacokinetics of CDSA-based materials reveal trends that challenge nanoparticle-derived assumptions, opening new opportunities to design anisotropic, degradable nanomedicines with predictable *in vivo* behaviour. Because organ distribution and systemic fate strongly shape therapeutic index and off-target toxicity, understanding how structural parameters influence biodistribution is critical (Fig. 4c and d).^129^ In 2024, Kempe *et al.* probed how corona chemistry and the site of drug conjugation on polymeric nanorods – prepared by heat-induced, “living CDSA” – affect pharmacological performance and biodistribution-related outcomes. They engineered POx/POzi-based nanorods bearing mycophenolic acid conjugates at different positions and compared variants with distinct hydrophilic coronas, while physicochemical characterisation confirmed uniform rod morphology with tuneable lengths. *In vitro* and formulation studies showed that corona hydrophilicity and conjugation site modulated drug release kinetics and interactions with serum components. Although their study emphasised release behaviour and therapeutic activity, the authors highlight that corona sterics and surface chemistry are expected to alter circulation persistence and organ accumulation (*e.g.*, hepatic clearance *vs* prolonged blood residence), thereby influencing both efficacy and off-target effects. Collectively, these results underscore that, for CDSA-derived nanorods, subtle changes in surface chemistry and drug attachment translate into meaningful differences in systemic distribution and therapeutic outcome – a crucial consideration when designing nanomedicines for controlled delivery.^130^

The *in vivo* behaviour of CDSA-derived nanostructures is central to their therapeutic performance. Their residence time in the bloodstream, patterns of organ uptake, and clearance routes are shaped by features such as surface chemistry, hydrophilic–hydrophobic balance, and particle geometry. These parameters collectively dictate delivery efficiency as well as unintended tissue exposure.^131,132^ As margination is an integral part of circulation, it refers to the movement of nanoparticles toward the endothelial walls within blood vessels, influencing their ability to interact with target cells and tissues. Stenzel and colleagues analysed how two-dimensional, platelet-like nanoparticles distribute within flowing media relative to spherical counterparts. By combining fluorescence imaging with SAXS measurements, they quantified particle positioning under various shear conditions and found that the flattened geometries consistently migrated toward vessel walls more effectively than spheres. This pronounced near-wall enrichment suggests that platelet-like carriers could offer advantages for vascular-targeted delivery by promoting stronger endothelial engagement and localized therapeutic action (Fig. 4e–g).^8^

Recent advances in biodegradable crystalline polymers, including polycarbonates and polypeptoids, allow CDSA to generate nanocarriers with controlled degradation profiles, enabling long-acting delivery and implantable systems.^133–135^ Understanding how self-assembled nanofibers break down in physiological settings is key for creating biodegradable carriers and implantable systems. Street and co-workers examined the enzymatic degradation of PFTMC nanofibers *vs.* nanospheres, focusing on how shape and crystallinity affect degradation behaviour. Using *Thermomyces lanuginosus* lipase, dynamic light scattering (DLS) measurements showed that nanofibers eroded at roughly one-third the rate of nanospheres, as a consequence of their more crystalline cores and reduced accessible surface. Even at enzyme levels relevant to biological conditions, nanofiber degradation proceeded through surface erosion, occurring primarily at the core-corona boundary where chain folds reside.^131^ Beyond extracellular or enzyme-mediated degradation, intracellular degradability is also an important advantage of biodegradable CDSA systems. Ganda *et al.* showed that self-assembled two-dimensional poly(ε-caprolactone) (PCL) platelet particles prepared by living CDSA were taken up by macrophages, trafficked through the endo/lysosomal pathway, and ultimately degraded completely in lysosomes.^136^ This finding highlights that CDSA-derived nanostructures can retain precise morphology during preparation and delivery while still undergoing breakdown after cellular internalisation. More broadly, because many CDSA systems are based on biodegradable semicrystalline polymers, such as PCL and PLLA, these carriers offer the attractive possibility of combining structural precision with degradation at the biological destination, although the degradation pathway and rate will depend on the polymer used and the intracellular environment. These observations indicate that CDSA enables the rational design of nanomedicines with predictable circulation, organ-specific accumulation, and controlled degradation, allowing a shift from empirically optimised carriers toward architecturally informed therapeutic strategies.

### Therapeutic delivery platforms: drug and gene transport

CDSA-derived nanocarriers provide architecture-dependent control over drug and gene encapsulation, release, and cellular delivery.^137^ However, the same structural features that make CDSA-derived particles attractive as nanocarriers also impose important constraints on cargo loading. In particular, the crystalline core can limit the direct encapsulation of hydrophobic small molecules relative to more amorphous carriers, while loading within the corona may alter hydrophilicity, colloidal stability, and biological interactions. As a result, CDSA-based delivery systems often rely on alternative strategies, including cargo incorporation in functional corona domains,^138^ localisation at interfacial or less crystalline regions,^139^ or covalent drug conjugation,^130^ each involving distinct trade-offs in loading capacity, release behaviour, and carrier stability. Their tuneable structure enhances cargo protection, reduces off-target effects, and supports stimulus-triggered release.^138,140^ Tong *et al.* engineered 2D platelet micelles to encapsulate hydrophobic DOX within P4VP domains, achieving minimal drug leakage at physiological pH and enhanced release under acidic tumour-like conditions, enabling efficient intracellular delivery.^141^ Similarly, the Manners group demonstrated that length-tunable polymer nanofibers serve as high-capacity, aggregation-resistant carriers for DNA, with shorter fibers (<100 nm) achieving superior transfection efficiency while maintaining high cell viability. Together, these examples illustrate how controlling nanocarrier morphology *via* CDSA can optimize cargo protection, cellular uptake, and stimulus-responsive release, offering promising platforms for precision drug and gene delivery.^142^ These examples underscore how CDSA transforms nanocarriers into architecturally programmable therapeutics, linking precise structural engineering with functional performance in complex biological environments.

### Energy transport and catalytic function

CDSA offers a structural paradigm for engineering nanostructures with precise control over crystalline order, interfaces, and defect minimisation, enabling predictive design of materials for energy conversion and catalysis. Unlike conventional polymer assemblies, CDSA generates monodisperse, architecturally defined nanostructures capable of long-range exciton diffusion and controlled catalytic environments.^2,143,144^

### Exciton and charge transport

One of the clearest demonstrations of how CDSA influences functional performance lies in its ability to modulate exciton migration pathways. Uniform 1D and 2D CDSA nanostructures facilitate long-range exciton transport *via* ordered π-stacked domains, a prerequisite for high-performance photovoltaics, photocatalysis, and optoelectronics.^145–147^

**Fig. 5:**
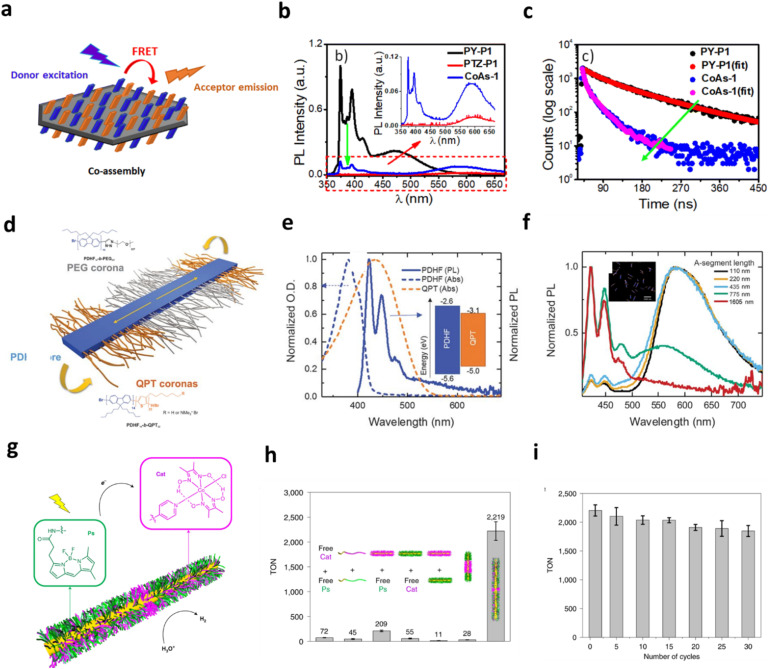
Architecturally programmed CDSA nanostructures for directed energy transport and catalytic activity. (a) Conceptual illustration of Förster resonance energy transfer arising from crystallisation-driven co-assembly of PY-PLLA and PTZ-P1 on two-dimensional platelet surfaces; (b) emission profiles of PY-P1, PTZ-P1, and their equimolar co-assembled structures in isopropanol (*λ*_ex_ = 337 nm, slit = 1 nm/1 nm), along with (c) corresponding time-resolved fluorescence decay curves for PY-P1 and the PY-P1/PTZ-P1 co-assembly (CoAs-1), evidencing efficient exciton coupling. Adapted from ref. 146 with permission from the Royal Society of Chemistry; (d) structural design of segmented B-A-B nanofibers incorporating spatially separated donor and acceptor domains; (e) steady-state absorption and photoluminescence spectra of the QPT homopolymer and unsegmented PDHF nanofibers, together with (f) normalized PL spectra of segmented PDHF nanofibers with different A-segment lengths; inset: LSCM image of uniform segmented nanofibers with a PDHF core (blue) and terminal QPT-corona segments (orange) in THF/MeOH (1 : 1). Excitation was at 380 nm. Adapted with permission from ref. 151; (g) schematic representation of photocatalytic Cat/Ps blend nanofibers for hydrogen evolution, comprising a crystalline PFS core and corona-tethered catalyst and photosensitizer units; (h) hydrogen evolution performance of Cat/Ps nanofibers after 5 h of irradiation in MeOH, benchmarked against control systems, and (i) TONs of the recycling runs of the blend photocatalytic system in MeOH. Adapted with permission from ref. 153.

A representative example arises from recent studies on chromophore-terminated PLLA that undergo CDSA into discrete 2D lozenge-shaped platelets. In these systems, polar or π-conjugated dyes appended to the PLLA chain ends are forced by lamellar crystallisation to occupy defined surface sites on the platelet faces, promoting controlled dipolar ordering and π–π stacking between neighbours. Co-assembly of donor- and acceptor-terminated PLLAs produces mixed platelets in which chromophores are held at nanometre separations across the 2D surface, enabling highly efficient Förster resonance energy transfer (FRET), with reported efficiencies approaching the high tens of percent for appropriately matched dye pairs over ∼10 nm length scales. Moreover, the presence of polar end groups was shown to enhance colloidal stability and uniformity of the platelets compared with purely hydrophobic termini, illustrating how CDSA affords nanoscale programmability of both spatial dye arrangement and resultant photophysical coupling – an organisational precision difficult to reproduce with non-crystalline or amorphous assemblies (Fig. 5a–c).^146^

Exciton transport in conjugated polymers is often limited by incoherent hopping, restricting diffusion to nanometer-scale distances. Achieving longer-range transport over hundreds of nanometers requires precise control over nanostructure size, morphology, and crystalline order.^148–150^ In this context, Manners, Friend, and co-workers prepared organic semiconducting nanofibers with a crystalline poly(di-*n*-hexylfluorene) (PDHF) core, polyethylene glycol corona, and polythiophene end blocks, which enabled efficient exciton transfer along the interchain π–π stacking direction from the PDHF core to lower-energy polythiophene segments, yielding diffusion lengths beyond 200 nm and a diffusion coefficient of 0.5 cm^2^ s^−1^ (Fig. 5d–f).^151^ Building on this, Rao *et al.* employed living CDSA to fabricate highly ordered poly(3-hexylthiophene) (P3HT) nanofiber films, achieving a regime of transient exciton delocalisation and markedly extended transport. In these systems, excitons coupled to vibrational modes to access spatially extended states, resulting in diffusion lengths of 300 ± 50 nm and diffusion coefficients of 1.1 ± 0.1 cm^2^ s^−1^, while energetic disorder was substantially reduced relative to regioregular P3HT.^152^ PXRD and low-dose scanning electron diffraction revealed well-packed crystalline domains with long-range orientational order and an average chain alignment persistence length of ∼80 nm. The living CDSA process, by promoting epitaxial growth from seed micelles and selective incorporation of defect-free polymer chains, minimised structural distortions and produced nanofibers with superior crystalline order compared to conventional spin-coating methods, establishing a clear link between nanostructural precision and enhanced exciton transport.

The above studies establish a design principle: CDSA enables exciton and charge transport by controlling architecture (core crystallinity, chain packing, and interface geometry) rather than relying solely on chemical modifications.

### Structural control of photophysical and optoelectronic properties

Beyond energy migration, optical and electronic properties are encoded *via* structural parameters such as fiber length, platelet thickness, and corona composition.^86,87^ This level of structural precision links static morphology to dynamic optoelectronic properties. For example, the controlled co-crystallisation of different π-scaffold-decorated PLLA chains leads to platelets with emergent photophysical characteristics, where dipole–dipole alignment and 2D packing govern emission spectra, exciton lifetime, and energy funneling efficiency.^89^ Similarly, the optical signatures of P3HT nanofibers – such as suppressed low-energy absorption (<1.7 eV) and reduced sub-bandgap disorder – directly reflect improvements in crystalline coherence and chain alignment achieved through living CDSA. These observations demonstrate the broader principle that CDSA enables photophysical design rules based on nanostructure geometry, where the arrangement of chromophores is encoded during crystallisation rather than imposed post-assembly. As a result, CDSA offers a pathway to organic materials whose optical properties are not empirically optimised but architecturally programmed.

The He group reported the fabrication of well-defined, size-controlled 2D diamond-shaped micelles using poly(*p*-phenylenevinylene) (PPV)-based block copolymers. The self-assembly of these nanostructures was guided by π–π stacking interactions, which enabled spatial separation of the semiconducting PPV cores from the insulating corona chains. These uniform 2D micelles were subsequently incorporated into vertical tunnelling devices, demonstrating an on–off current ratio exceeding 10^4^ and a peak on-state current density of 6000 A cm^−2^ as measured by conductive atomic force microscopy (AFM). Additionally, the compressible P2VP corona was exploited to develop a pressure-sensitive tunnelling sensor, where external mechanical force modulated the insulating layer thickness and thus the vertical tunnelling current. The sensor exhibited reversible and stable current responses, reaching 3 nA under a maximum applied force of 148 nN, with excellent reproducibility and minimal performance degradation over multiple cycles. This work highlights how CDSA-derived 2D micelles can be precisely engineered for both electronic and mechanoresponsive device applications.

### Catalysis

CDSA-derived nanostructures provide spatially ordered scaffolds that improve catalytic efficiency, substrate accessibility, and selectivity. These nanostructures act either as standalone catalysts or as scaffolds that stabilise and organise catalytic species, optimising substrate access, intermediate handling, and overall reaction pathways.^154–156^ In some cases, block copolymer assemblies even possess intrinsic catalytic activity, usually localised in the corona, allowing them to participate directly in chemical transformations.^157–161^

CDSA has proven particularly powerful for constructing π-conjugated nanofibers with well-defined electronic properties for photocatalysis. Feng *et al.* prepared donor–acceptor co-oligomer nanofibers, using benzothiadiazole (BT) or dibenzo[*b*,*d*]thiophene 5,5-dioxide (FSO) units attached to the two ends of an oligo(*p*-phenylene ethynylene) heptamer (*e.g.*, BT-OPE_7_-BT, FSO-OPE_7_-FSO), where the terminal electron-deficient motifs promoted ordered π–π stacking.^162^ Assembly in ethanol *via* CDSA enabled length regulation through self-seeding and seeded growth, yielding crystalline π-conjugated nanofibers (CPNFs) with improved charge separation and enhanced photocatalytic oxidation of sulfides and amines. Their study underscored how terminal group design and nanofiber order dictate charge transport and catalytic behaviour. In parallel, Tian *et al.* reported a recyclable photocatalytic core–shell nanofiber integrating a cobalt catalyst and a BODIPY photosensitiser for visible-light-driven hydrogen production (Fig. 5g–i).^153^

Beyond intrinsic catalysis, CDSA-derived assemblies serve as robust platforms for immobilising catalytic nanoparticles. Qiu *et al.* created micellar brushes on silicon substrates using PFS-*b*-P2VP seeds, growing fiber-like brushes up to ∼1 mm long.^163^ Subsequent decoration with ∼15 nm Au nanoparticles produced hybrid surfaces that efficiently catalysed borohydride reduction of 4-nitrophenol, maintaining high activity and recyclability over 15 cycles. This strategy also enabled their integration into flow systems for multistep cascade reactions, such as glucose oxidation coupled with peroxidase-like 3,3′,5,5′-tetramethylbenzidine (TMB) oxidation, illustrating the adaptability of CDSA-derived brushes for continuous, complex transformations.^155^ Complementing these approaches, Zhang *et al.* developed crystalline π-conjugated polyfluorene nanofibers functioning as antenna systems for Co(ii) porphyrin photocatalysts, boosting solar-driven hydrogen generation.^164^ Dimensional control *via* seeded CDSA enhanced exciton diffusion (>200 nm) and exciton transfer efficiency, yielding hydrogen evolution rates of ∼65 mmol h^−1^ g^−1^, turnover numbers >450, and quantum yields of 0.4% under <405 nm irradiation. Together, these examples demonstrate how CDSA's control over nanostructure morphology, length, and composition translates directly into enhanced energy conversion and catalytic function, positioning CDSA as a generalisable platform for advanced optoelectronic and catalytic materials.

## Conclusions

CDSA has evolved into a uniquely powerful strategy for the construction of 1D and 2D nanomaterials with a level of precision that bridges molecular design and mesoscale function. By harnessing directional crystallisation as an architectural principle, CDSA enables deterministic control over nanoparticle size, shape, internal segmentation, and crystallinity – features that collectively underpin the remarkable functional performance observed across optical, mechanical, biological, energy, and catalytic applications.

A defining strength of CDSA lies in its predictability. Unlike many self-assembly processes that rely on empirical optimisation, living and seeded CDSA provide a framework in which nanostructure dimensions and composition can be forecast from molecular inputs and processing conditions. This predictive capability is increasingly amplified by the integration of continuous flow processing, real-time analytics, and non-equilibrium crystallisation control, transforming CDSA from a batch-based laboratory technique into a scalable, programmable manufacturing platform.

Across optoelectronic materials, mechanical reinforcement, biomedical delivery, catalysis, and energy conversion, a consistent theme emerges: CDSA enables function to be dictated by nanoscale architecture with a level of determinism rarely achieved in polymeric systems. The emerging applications of CDSA are therefore not defined by entirely new end uses, but by the convergence and maturation of these structure–function relationships into predictable, design-led material platforms.^165,166^

In optical information storage and photonic systems, CDSA has progressed from proof-of-concept fluorescent barcodes to multi-dimensional encoding strategies that integrate spatial segmentation, emission colour, and temporal lifetime modulation. These advances suggest an evolution from static optical labels toward reconfigurable and information-dense soft photonic materials, where data density and addressability are encoded during assembly rather than imposed post-fabrication. As living CDSA and flow-enabled growth improve throughput and reproducibility, such systems may transition from single-particle demonstrations to scalable ensembles capable of device-level integration.

In mechanically functional materials, CDSA-derived 1D and 2D nanoparticles have already demonstrated clear advantages as reinforcing agents, adhesives, and friction modifiers, outperforming isotropic fillers through their controlled aspect ratio and crystalline rigidity. Emerging applications in this space point toward architected soft composites, in which nanostructure dimensions are deliberately matched to polymer network length scales, crack propagation pathways, or interfacial stress distributions. The ability to tune particle length, thickness, and surface chemistry independently suggests future materials where mechanical performance is programmed rather than empirically optimised.

Biomedical applications reveal perhaps the clearest trajectory from fundamental control to translational potential. Systematic studies on cellular uptake, immunomodulation, biodistribution, and degradation have established that CDSA-derived nanostructures act as geometry-defined biological entities, rather than generic carriers. Emerging opportunities therefore lie in exploiting CDSA assemblies as libraries of precisely defined probes to map biological response landscapes, and as therapeutic platforms where circulation time, tissue penetration, and immune activation are encoded through nanostructural design.

In energy transport and catalysis, CDSA has enabled unprecedented control over exciton diffusion, charge transport, and active-site organisation by minimizing defects and enforcing long-range order. The emerging direction in this area is the integration of CDSA-derived nanostructures into hierarchical energy and catalytic systems, where controlled nanoscale pathways are coupled with macroscopic device architectures. Flow-compatible CDSA and hybrid organic-inorganic assemblies further suggest routes toward continuous, modular energy and catalytic materials. Taken together, these developments indicate that the most significant emerging application of CDSA is not a single technology, but a general design framework: the use of crystallisation-defined polymer architectures as programmable functional elements. As synthetic control, flow processing, and real-time characterisation continue to advance, CDSA-derived materials are poised to transition from bespoke laboratory systems to broadly deployable platforms across multiple sectors.

Looking forward, several important challenges must still be addressed before CDSA can fully mature into a broadly deployable materials platform. Although continuous-flow processing has begun to overcome some practical limitations of batch CDSA, including improved reproducibility, thermal-history control, throughput, and seed preparation efficiency, it does not eliminate the underlying complexity of the assembly process. Important obstacles remain, including the need for more predictive design rules across broader polymer classes, improved control over nucleation and growth under non-equilibrium conditions, and better integration of CDSA with scalable polymer synthesis, downstream processing, and end-use fabrication. Addressing these issues will be essential if CDSA is to move beyond highly optimised model systems toward more general and application-ready materials platforms.

The next phase innovations in the field will depend on tackling application-specific shortcomings more directly. In nanomedicine, for example, the crystalline core that gives CDSA-derived particles their structural precision can also restrict conventional drug loading, meaning that future progress will require strategies that decouple cargo incorporation from core crystallinity. Promising directions include cargo loading in corona or corona-associated domains, localisation at interfacial or less-crystalline regions, covalent conjugation approaches, and the development of multicompartment or core-shell-corona-like CDSA particles in which structural stability and payload capacity are spatially separated. More broadly, expanding the palette of crystallisable polymers – particularly biodegradable, stimuli-responsive, and electronically active systems – together with advances in in-line characterisation, machine learning-guided process optimisation, and autonomous processing, will help to transform CDSA from a powerful assembly concept into a more versatile and widely deployable design framework for soft functional materials.

## Author contributions

The manuscript was written with contributions from both authors.

## Conflicts of interest

There are no conflicts of interest to declare.

## Data Availability

No primary research results, software or code have been included, and no new data were generated or analysed as part of this review.
